# Increased Expression of Fatty-Acid and Calcium Metabolism Genes in Failing Human Heart

**DOI:** 10.1371/journal.pone.0037505

**Published:** 2012-06-06

**Authors:** Vanessa García-Rúa, Manuel Francisco Otero, Pamela Virginia Lear, Diego Rodríguez-Penas, Sandra Feijóo-Bandín, Teresa Noguera-Moreno, Manuel Calaza, María Álvarez-Barredo, Ana Mosquera-Leal, John Parrington, Josep Brugada, Manuel Portolés, Miguel Rivera, José Ramón González-Juanatey, Francisca Lago

**Affiliations:** 1 Laboratory of Cellular and Molecular Cardiology, Santiago Institute of Biomedical Research (IDIS), University of Santiago de Compostela Clinical Hospital (CHUS), Santiago de Compostela, Spain; 2 Laboratory 10, Santiago Institute of Biomedical Research (IDIS), University of Santiago de Compostela Clinical Hospital (CHUS), Santiago de Compostela, Spain; 3 Department of Clinical Chemistry, University of Santiago de Compostela Clinical Hospital (CHUS), Santiago de Compostela, Spain; 4 Department of Cardiology, University of Santiago de Compostela Clinical Hospital (CHUS), Santiago de Compostela, Spain; 5 Unit of Biostatistical Research, University of Santiago de Compostela, Santiago de Compostela, Spain; 6 Department of Pharmacology, Oxford University, Oxford, United Kingdom; 7 Cardiology Department, Thorax Institute, Hospital Clinic, Barcelona, Spain; 8 La Fe University Hospital, Valencia, Spain; University of Otago, New Zealand

## Abstract

**Background:**

Heart failure (HF) involves alterations in metabolism, but little is known about cardiomyopathy-(CM)-specific or diabetes-independent alterations in gene expression of proteins involved in fatty-acid (FA) uptake and oxidation or in calcium-(Ca^2+^)-handling in the human heart.

**Methods:**

RT-qPCR was used to quantify mRNA expression and immunoblotting to confirm protein expression in left-ventricular myocardium from patients with HF (n = 36) without diabetes mellitus of ischaemic (ICM, n = 16) or dilated (DCM, n = 20) cardiomyopathy aetiology, and non-diseased donors (CTL, n = 6).

**Results:**

Significant increases in mRNA of genes regulating FA uptake (CD36) and intracellular transport (Heart-FA-Binding Protein (HFABP)) were observed in HF patients vs CTL. Significance was maintained in DCM and confirmed at protein level, but not in ICM. mRNA was higher in DCM than ICM for peroxisome-proliferator-activated-receptor-alpha (PPARA), PPAR-gamma coactivator-1-alpha (PGC1A) and CD36, and confirmed at the protein level for PPARA and CD36. Transcript and protein expression of Ca^2+^-handling genes (Two-Pore-Channel 1 (TPCN1), Two-Pore-Channel 2 (TPCN2), and Inositol 1,4,5-triphosphate Receptor type-1 (IP3R1)) increased in HF patients relative to CTL. Increases remained significant for TPCN2 in all groups but for TPCN1 only in DCM. There were correlations between FA metabolism and Ca^2+^-handling genes expression. In ICM there were six correlations, all distinct from those found in CTL. In DCM there were also six (all also different from those found in CTL): three were common to and three distinct from ICM.

**Conclusion:**

DCM-specific increases were found in expression of several genes that regulate FA metabolism, which might help in the design of aetiology-specific metabolic therapies in HF. Ca^2+^-handling genes TPCN1 and TPCN2 also showed increased expression in HF, while HF- and CM-specific positive correlations were found among several FA and Ca^2+^-handling genes.

## Introduction

Human heart failure (HF) has been described as a hyperadrenergic state leading to increased plasma levels of free fatty acids (FFA), which in turn cause a range of metabolic dysfunctions including aberrant FA uptake and oxidation in the myocardium [1]. Therapeutic modulation of FA metabolism [1], [2], stimulation of glucose metabolism [3] and selective targeting of sarcolemmal transporters of FA or glucose, depending on presence or absence of diabetes [4] are all currently under consideration. The putatively adaptive switch from predominantly FA to predominantly glucose oxidation via the Randle cycle during progression to myocardial failure has received increasing attention [3], [5]–[7]. However, mechanisms linking alterations in FA uptake and oxidation *per se* with HF in humans, particularly in relation to aetiology and treatment, remain poorly understood.

One consistent finding in HF patients is an increase in plasma levels of Heart Fatty-Acid-Binding Protein (HFABP) which can be substantial [8]–[10]. In light of its role in intracellular transport to the mitochondria where FAs undergo β-oxidation [11], it is of note that expression levels of HFABP within the myocardium have not previously been studied in HF patients. Potential alterations in human HF between cardiac HFABP and its downstream enzymes of FA oxidation are thus unknown. An important regulator of HFABP and several subsequent steps in FA utilisation, peroxisome-proliferator-activated-receptor-alpha (PPARA) [12], [13], has also been found to have altered expression in human HF [14]–[16].

HF-related changes in myocardial gene expression have also been found at various points in the FA uptake and oxidation pathways in humans and animal models for the following genes: Cluster-of-Differentiation 36 (CD36, also known as Fatty-Acid Translocase (FAT) and an important FA transporter across the plasma and outer mitochondrial membranes) [5], [17]; Cardiac Carnitine Palmitoyl-Transferase-1 (CPT-1, the other principal outer mitochondrial membrane FA transporter) [15], [18], [19]; and Long-Chain Acyl-CoA Dehydrogenase (LCAD, a key enzyme of β-oxidation) [18], [20]–[22].

PPARA and LCAD are also known to be regulated by PPAR-gamma coactivator-1-alpha (PGC1A) [23], while altered mitochondrial function has been associated with excess levels of plasma FFA in HF patients [24], and in rodent models with excess FA levels not only in plasma but also within the cardiomyocyte [25]–[27]. However, many discrepancies still exist in the findings of studies on alterations in the FA uptake-oxidation pathway as a whole in human HF. This is probably due to HF having been viewed as a single pathological end-point, rather than as different aetiology-specific diseases [28], [29].

Aberrant lipid metabolism can also disrupt intracellular calcium (Ca^2+^) homeostasis – specifically Ca^2+^ cycling and inositol-3-phosphate receptors (IP_3_Rs) levels – and cause endoplasmic reticulum (ER) stress [30]–[33], one of whose main markers is known to be the protein CHOP (CCAAT/enhancer-binding-protein-homology protein) [34], [35]. Altered expression of IP_3_Rs has previously been identified in cardiac hypertrophy patients [36]. The two-pore channels TPCN1 and 2 have been recently identified as a novel class of endo-lysosomal Ca^2+^ channels and likely receptors for NAADP, nicotinic acid adenine dinucleotide phosphate, the most potent Ca^2+^ mobilizing messenger known [37], [38], [39].

Our purpose was to look for changes in the expression of proteins involved in FA uptake and oxidation or in Ca^2+^-handling in the failing human heart, with the aim of defining aetiology-specific differences between ischaemic and dilated heart failure patients.

## Materials and Methods

### Tissue Samples, and Demographic and Clinical Parameters

Transmural left-ventricular myocardial biopsies were obtained from explanted human hearts from 16 patients with ICM and 20 patients with DCM, all without diabetes mellitus, in end-stage HF and undergoing heart transplantation, as previously described [40], [41]. Cardiomyopathy groups were defined according to the following clinical criteria: ICM: LVEF <40% with coronary artery damage on angiogram (the majority having suffered a previous MI); DCM: LVEF <40% with intact coronary arteries, no previous MI, and dilated non-hypertrophic left ventricle on echocardiography (LVDD >55 mm [40], [41]; Table 1). Non-diseased donor hearts in cardioplegic arrest for a mean time of two and a half hours, deemed unsuitable for transplant due to blood-type or size incompatibility, were used to obtain control myocardial tissue (CTL, n = 6; Table 2). The atrial tissue necessary for the standarization of some of the antibodies used in Western blot was obtained as pieces of right atrial appendage excised to allow catheterisation of the right atrium during surgery requiring cardiopulmonary bypass (tissue which is usually discarded).

**Table 1:** Patient characteristics according to heart failure aetiology.

	ICM (n = 16)	DCM (n = 20)
Age (yr), mean±SD	53.6±8.1	46.5±11
Gender male, n (%)	16 (100)	17 (85)
NYHA class (1–4), mean±SD	3.61±0.3	3.44±0.5
BMI (kg/m^2^), mean±SD	25.5±4.0	25.1±4.6
LVEF (%), mean±SD	23.2±5.6	19.6±7.6
LVESD (mm), mean±SD	53.4±7.4	69.8±9.4
LVEDD (mm), mean±SD	61.7±7.3	78.3±8.8
LV mass (g), mean±SD	258.7±54.2	390.8±116.4
Duration of disease (m), mean±SD	37.8±46.4	69.5±52.6

Duration of disease: from diagnosis to heart transplant; BMI: body mass index; DCM: dilated cardiomyopathy; ICM: ischaemic cardiomyopathy; LVEF: left ventricular ejection fraction; EDD: end diastolic diameter; ESD: end systolic diameter; NYHA: New York Heart Association.

**Table 2:** Characteristics of control (CTL) group.

Donor	Age	Gender	Cause of death	LVEF
1	16	Female	RTA	>50
2	58	Male	UNK	>50
3	56	Male	RTA	>50
4	56	Male	CVA	>50
5	54	Female	CVA	>50
6	51	Male	UNK	>50

CVA: cerebrovascular accident; LVEF: left ventricular ejection fraction (%, value assumed); UNK: data not available; RTA: road traffic accident.

All tissues were obtained with signed informed consent of patients. The project was approved by the local Ethics Committee (Biomedical Investigation Ethics Committee) and conducted in accordance with the guidelines of the Declaration of Helsinki.

### Real-time PCR

RNA was extracted using a NucleoSpin kit, according to the manufacturer’s instructions (Macherey-Nagel, Germany). For relative quantification we performed a reverse-transcription reaction with 1 µg of RNA using a First Strand kit (Superarray Bioscience Corp, MD, USA). RNA quality and quantity was determined using a NanoDrop (Thermo Scientific, Spain) spectrophotometer. Next, a real-time PCR reaction was performed using the Superarray master mix provided, and 1 µl of cDNA in each well, for the following specific human primers: TPCN1∶86 bp, PPH08675A, reference position 2682 RefSeq **NM_017901.4**; TPCN2∶124 bp, PPH13927 reference position 1550 RefSeq Accession: **NM_139075.3**; GAPDH: 175 bp, PPH00150E reference position 1287–1310 GenBank **NM_002046.3**. Amplification conditions were: 95°C for 10 min, followed by 40 cycles of 95°C for 15 s and 60°C for 60 s. All Superarray primers were pre-optimised by the manufacturer.

A real-time PCR reaction was performed using the Solaris qPCR Gene Expression Master Mix with LOW ROX premixed, and 1 µL of total cDNA in each well, for the following specific human primers: GAPDH: GCCTCAAGATCATCAGCAATG (forward) and CCTCCACGATACCAAAGTTGTC (reverse), Probe (GCCAAGGTCATCCATGA), RefSeq Accession **NM_002046**; IP3R1: TGGGGCACAACATCTAC (forward) and CTTTGTTATGCCGAGCCA (reverse), Probe (ATTAGCCCATCAGTTGGC), RefSeq Accession **NM_002222**; **NM_001099952;** PPARA: CCAGTATTTAGGAAGCTGTCC (forward) and TGAAAGCGTGTCCGTGAT (reverse), Probe (CTCAGATGGCTCGGTCA), RefSeq Accession **NM_032644**; PGC1A: AATTGAAGAGCGCCGTGT (forward) and AACCATAGCTGTCTCCATC (reverse), Probe (AGTAAATCTGCGGGATG), RefSeq Accession **NM_013261**; CPT1B: ACTGCTACAACAGGTGGTT (forward) and TCTGCATTGAGACCCAACTG (reverse), Probe (ATTTCCTTCAAGAATGGCC), RefSeq Accession **NM_152247**; **NM_152245**; **NM_152246**; **NM_004377**; CD36: GTTGCCATAATCGACAC (forward) and GCAGTGACTTTCCCAATAGG (reverse), Probe (GGTAAAAGGAATCTGTCC), RefSeq Accession **NM_001001548**; **NM_001001547**; **NM_000072**; **NM_001127443**; **NM_001127444** and LCAD: CTTCCACAGGAAAGGCTGTT (forward) and CTGCTAATTTATGTTGCACTG (reverse), Probe (GTTGCTCACCTACAGACAGT), RefSeq Accession **NM_001608.**

Using primers designed with the Beacon Designer program (v3.1, Premier Biosoft International, CA, USA) and pre-optimised conditions, we also performed one-step real-time PCR with the Brilliant II SYBR Green QRT-PCR Master Mix Kit (Stratagene, CA, USA) and the same quantity of total RNA in each well (5 ng), for the following specific human primers: CHOP: CAGAACCAGCAGAGGTCACA (forward) and TCACCATTCGGTCAATCAGA (reverse), 210 bp, RefSeq Accession **NM_004083.4**; HFABP: CAGCAGATGACAGGAAGG (forward) and CCGATTGGCAGAGTAGTAG (reverse), 218 bp, RefSeq Accession **NM_004102.3**; GAPDH: AAGGTGAAGGTCGGAGTC (forward) and CCTGGAAGATGGTGATGG (reverse), 229 bp, RefSeq Accession **NM_002046.3**. Amplification conditions were: reverse-transcription reaction: 50°C for 30 min and 95°C and 10 min; polymerase-chain reaction: 40 cycles of 95°C for 40 s, 56°C for 60 s, and 72°C for 40 s. Efficiency curves of 90–110% were obtained.

Results were analyzed using the MxPro v4 software (Stratagene, CA, USA). PCR experiments for each gene in each donor and patient were performed in duplicate and the mean value used to calculate fold-change in expression (2^−ΔΔCt^, where ΔC_t_ = mean (GOI-Ref) value at threshold cycle; GOI: gene of interest; Ref: reference gene). <1% of duplicate experiments were repeated due to a threshold cycle ≥35 or difference in threshold cycles of >1. Experiments were standardised by use of a single pool of extracted RNA for each donor or patient and in each plate an internal reference gene (GAPDH) and an external single pool of human non-ventricular (atrial) RNA. A dissociation curve was obtained at the end of each experiment to confirm the PCR product.

### Immunoblotting

Individual genes showing HF- and CM-related changes in expression at the RNA level (HFABP, TPCN1, TPCN2, PPARA, IP_3_R1, CD36) were then tested at the protein level by immunoblotting. Tissue was lysed with Triton X-100 (1% buffered in 50 mmol/L Tris-HCl, 150 mmol/L NaCl, 5 mmol/L EDTA, 1 mmol/L phenylmethylsulphonylfluoride, 10 µg/ml leupeptin, 10 µg/ml aprotinin, 10 µg/ml trypsin inhibitor and 1 mmol/L NaVO_4_). Samples were subjected to SDS-PAGE under denaturing conditions on 8% (TPCN1, TPCN2), 15% (HFABP), 10% (PPARA, CD36) and 4–20% (IP_3_R1) acrylamide gels and electroblotted onto PVDF membranes (Amersham Pharmacia Biotech, Germany). Membranes were treated with the following primary antisera, either at room temperature for 2 h (anti-PPARA, TPCN1, TPCN2) or at 4°C overnight (HFABP, CD36): HFABP (1∶000; Santa Cruz Biotechnology, CA, USA); anti-CD36 (1∶1000; Pierce Biotechnology-Thermo Scientific, IL, USA); anti-TPCN1 (1∶1000) affinity-purified using a standard kit according to the manufacturer’s protocol (SulfoLink, Thermo Scientific UK) from rabbit anti-serum raised against specific TPCN1 antigenic human C-peptides; anti- TPCN2 (1∶100; Novus Biologicals, CO, USA); anti-PPARA (1∶1000; Pierce Biotechnology-Thermo Scientific, IL, USA); and anti-IP_3_R. Membranes were then incubated with horseradish-peroxidase-conjugated secondary antibody (1∶2000; Santa Cruz Biotechnology, CA, USA) and subjected to chemiluminescence detection (Millipore Corporate, MA, USA). Anti-GAPDH (1∶1000; Santa Cruz Biotechnology, CA, USA) was used as a loading control. Densitometric analyses were performed using a UVP EC3 Imaging System (Ultra-Violet Products Ltd, UK) and the Image J program (v1.43 q; Rasband 1997–2008).

### N-Glycosidase F (PNGase F) Pre-treatment for Identification of TPCN1 and TPCN2 Protein Bands in Western Blot

Ours is the first report that includes the identification by western blot of the protein bands corresponding to TPCN1 and TPCN2 in human myocardium. In order to standarize a protocol for the identification of both proteins in human cardiac tissue, and having into account that in other tissues TPCN1 and TPCN2 are known to be N-glycosidated [42], [43], we first performed several experiments that helped us to identify both proteins in our western blots. Briefly, and as described previously for HEK 293 cells [42], [43], atrial protein (extract supernatant containing 20 µg total protein) was incubated with or without 50 000 units/ml N-Glycosidase F (PNGase F, New England Biolabs, USA) before immunoblotting as described above. For TPCN2 the manufactureŕs protocol was followed, i.e. protein was previously denatured at 100°C for 10 min and then incubated in PNGase F at 37°C for 60 min; for TPCN1, the denaturing step was omitted and PNGase F incubation carried out for 120 min.

Comparing PNGase F pre-treated and non-pretreated atrial protein by immunoblot revealed different protein bands for both TPCN1 and TPCN2. Thus TPCN1 and TPCN2 expressed endogenously in human myocardium was shown to be N-glycosylated. Glycosylation of TPCN1 corresponded with bands of molecular weights of 130 and 100 kDa which disappeared after PNGase F pre-treatment, while a more prominent band of 95 kDa appeared probably due to the protein running at a lower molecular weight after deglycosylation with PNGase F treatment (data not shown). Glycosylation of TPCN2 corresponded with two bands of molecular weights approximately 100 and 85 kDa; while a band of approximately 80 kDa appeared probably due to the protein running at a lower molecular weight after deglycosylation with PNGase F treatment (data not shown).

### Statistical Analyses

We used a non-parametric approach because the patient population did not show a normal distribution for all demographic or clinical variables investigated (Shapiro-Wilk normality tests). For mRNA analysis, statistical significance of the differences between fold-changes in gene expression above baseline (2^−ΔΔCt^; hereafter referred to as gene expression levels) were tested using U Mann-Whitney, Kruskall-Wallis and post-hoc tests, for CTL, HF, ICM and DCM groups. For protein-level analysis, U Mann-Whitney or the Wilcoxon Sign Test was used. Spearman linear correlation coefficients were then calculated for gene expression levels, within each of the HF, ICM and DCM groups. SPSS v15.0 (SPSS Inc, IL, USA) and R 2.12.2 (R Development Core Team (2011) [44] were used. Significance was taken at p<0.05 in all but post-hoc U Mann-Whitney tests where p<0.016 was considered significant.

## Results

### HF- and Aetiology-related Changes in Expression of Individual Genes of Fatty Acid Metabolism

**Figure 1:**
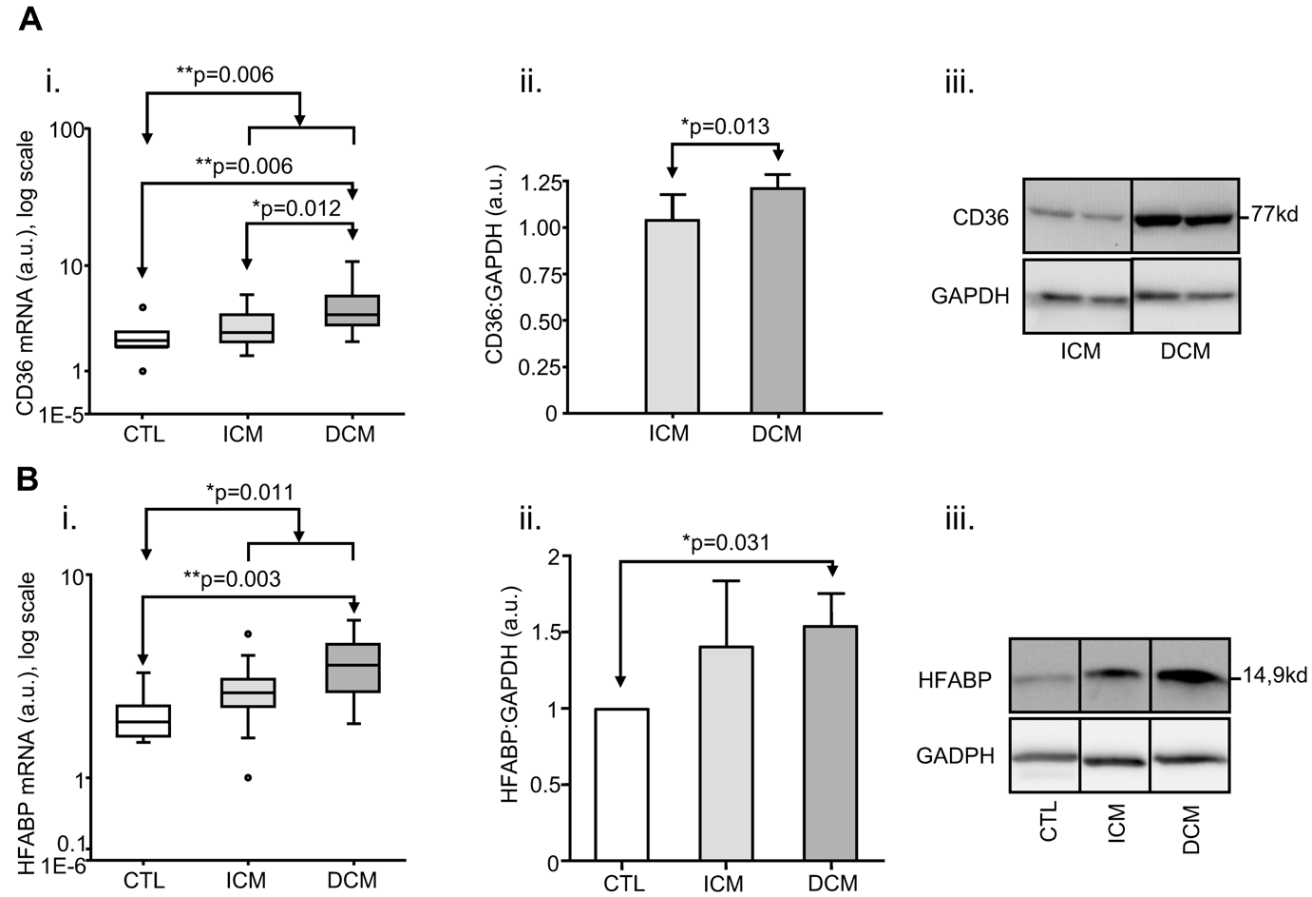
HF- and aetiology-related changes in expression levels of individual genes/proteins of fatty acid metabolism. **A**. Increased human myocardial expression of Cluster-of-Differentiation 36 (CD36) in heart failure as a whole (HF) and aetiological groups in absence of diabetes mellitus (ischaemic or dilated cardiomyopathy (ICM, DCM) and compared with non-diseased donors (CTL). **i**. box-plot of mRNA expression, showing a significant increase over CTL (n = 6) for HF (n = 36, Mann-Whitney p = 0.006) and DCM (n = 20, post-hoc p = 0.006), but not ICM (n = 16); mRNA was also more highly expressed in DCM than ICM (post-hoc p = 0.012); **ii**. densitometry analysis of protein expression from immunoblots, showing a significant increase over ICM (n = 7) for DCM (n = 7, p = 0.013); **iii**. representative immunoblot for ii. **B**. Increased human myocardial expression of Heart-Fatty-Acid-Binding Protein (HFABP) with respect to heart failure as a whole and aetiological groups in absence of diabetes mellitus. **i.** box-plot of mRNA expression, showing a significant increase over non-diseased donors (CTL, n = 6) for HF (n = 36, p = 0.011) and DCM (n = 20, p = 0.003), but not ICM (n = 16); **ii**. densitometry analysis of protein expression from immunoblots, showing a significant increase over CTL (n = 3) for DCM (n = 7, p = 0.031); **iii**. representative immunoblot for ii. (a.u.: arbitrary units).

In HF (n = 36) as a whole, mRNA expression for CD36 (p = 0.006) and HFABP (p = 0.011) was found to be increased relative to CTL (Table 3 and Fig. 1).

**Table 3:** Increases in myocardial gene expression in heart failure groups, according to cardiomyopathic aetiology.

Gene	Comparisons vs. CTL			ICM vs.DCM
	HF	ICM	DCM	
CD36	p = 0.006	ns	p = 0.006	p = 0.012
HFABP	p = 0.011	ns	p = 0.003	ns
PPARA	ns	ns	ns	p = 0.015
PGC1A	ns	ns	ns	p = 0.010
TPCN1	p = 0.018	ns	p = 0.015	ns
TPCN2	p = 0.001	p = 0.008	p = 0.001	ns
IP3R1	p = 0.035	ns	ns	ns

mRNA expression in heart failure (HF, n = 36) and aetiological groups of HF, compared with Control (CTL, n = 6) and directly between two groups: ICM (n = 16): ischaemic cardiomyopathy; DCM (n = 20): dilated cardiomyopathy. ns: not significant.

When considering CM-specific groups, mRNA expression of CD36 (p = 0.006) and HFABP (p = 0.003) was increased relative to CTL only in DCM (n = 20) but not in ICM (n = 16) (Table 3 and Fig.1).

Analysis by western blot of the protein levels of CD36 confirmed that expression of this protein was significantly higher (p = 0.013, Mann-Whitney test) in the cardiac tissue of patients with HF of dilated aetiology (DCM; n = 7) than in those of ischaemic aetiology (ICM; n = 7) (Fig. 1).

HFABP protein levels analysis by western blot confirmed that the expression of this protein was significantly increased only in HF patients of dilated aetiology (n = 6 DCM *vs* n = 3 CTL, p = 0.031) (Fig. 1).

**Figure 2:**
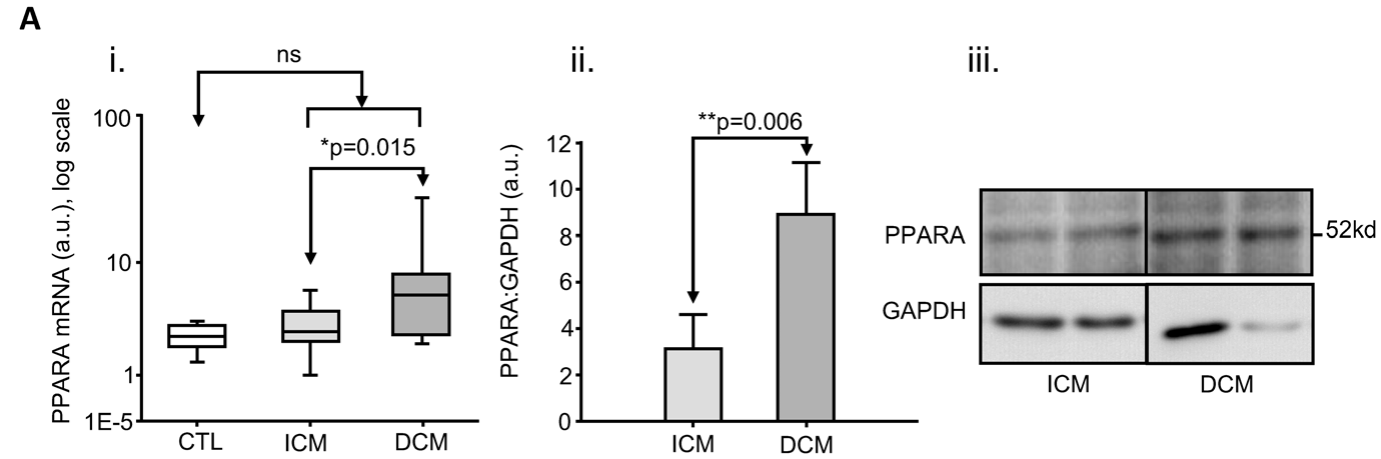
Different human myocardial expression of peroxisome-proliferator-activated-receptor-alpha (PPARA) between the two heart failure aetiological groups in absence of diabetes mellitus. i. box-plot of mRNA expression, showing a significant increase in DCM (n = 20) compared with ICM (n = 16, p = 0.015); ii. densitometry analysis of protein expression from immunoblots, showing a significant increase in DCM (n = 7) compared with ICM (n = 7, p = 0.006); iii. representative immunoblot for ii. (a.u.: arbitrary units).

Finally, a direct comparison between ICM (n = 16) and DCM (n = 20) using post-hoc tests showed mRNA of the following three genes to be more highly expressed in DCM than ICM: PPARA (p = 0.015), PGC1A (p = 0.010), CD36 (p = 0.012) (Table 3 and Figs.1 and 2).

DCM>ICM was confirmed at the protein level for PPARA (DCM n = 7 *vs* ICM n = 7, p = 0.006) (Fig.2).

We did not find significant changes in mRNA expression levels for the following genes (data not shown): CPT1B, LCAD, CHOP.

### HF- and Aetiology-related Changes in Expression of Ca^2+^-Handling Genes

**Figure 3:**
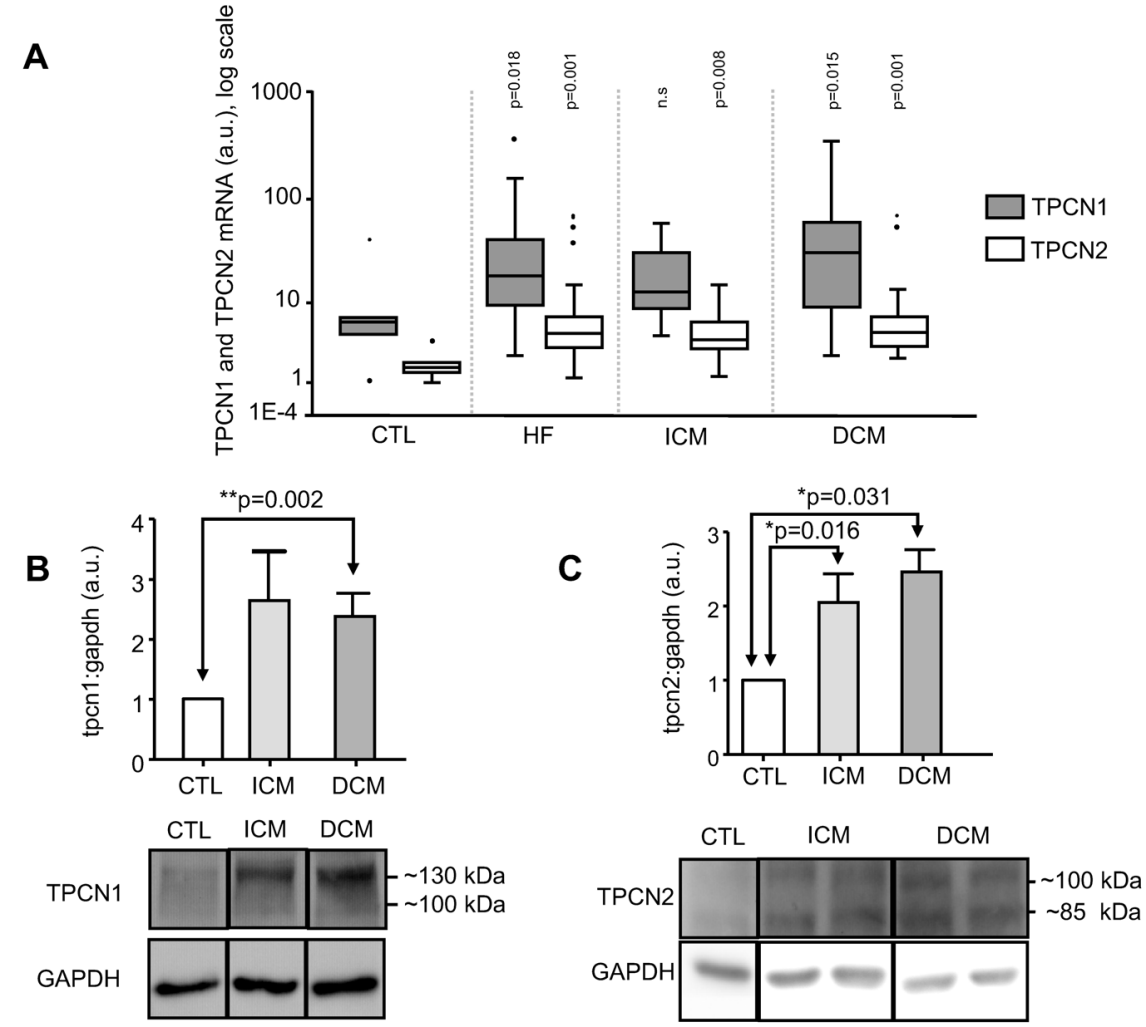
Increased human myocardial expression of Two-Pore Calcium channels TPCN1 and TPCN2 in heart failure as a whole (HF) and aetiological groups (ischaemic or dilated cardiomyopathy, ICM, DCM) and compared with non-diseased donors (CTL). A. Box-plot of TPCN1 and TPCN2 mRNA expression, showing for TPCN1 a significant increase over CTL (n = 6) for HF (n = 36, p = 0.018) and DCM (n = 20, p = 0.015); and for TPCN2 a significant increase over CTL (n = 6) for HF (n = 36, p = 0.001), ICM (n = 16, p = 0.008) and DCM (n = 20, p = 0.001). B. Densitometry analysis and representative immunoblot of TPCN1 protein expression showing a significant increase over CTL (n = 4) for DCM (n = 13, p = 0.002). C. Densitometry analysis and representative immunoblot of TPCN2 protein expression showing a significant increase over CTL (n = 4) for both ICM (n = 8, p = 0.016) and DCM (n = 6, p = 0.031). (a.u.: arbitrary units).

**Figure 4:**
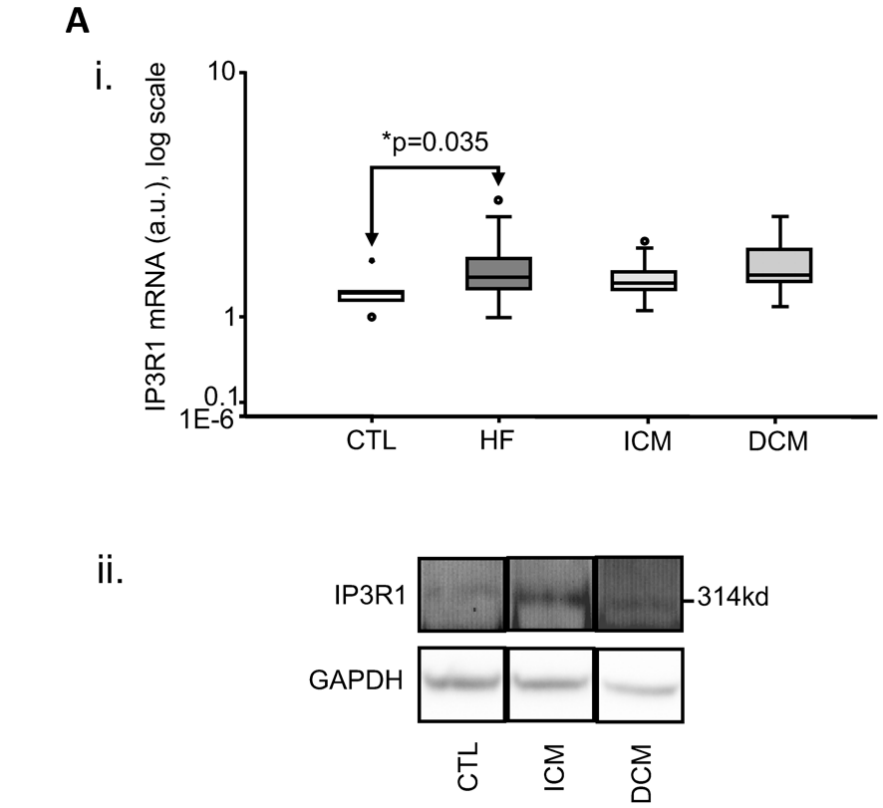
Increased human myocardial expression of Inositol-3-Phosphate Receptor sub-type 1 (IP_3_R1) in heart failure as a whole (HF, n = 36). i. Box-plot of mRNA expression, showing a significant increase over CTL (n = 6, p = 0.035); ii. representative immunoblot for protein expression of IP_3_R1. (a.u.: arbitrary units).

In HF (n = 36) as a whole, mRNA expression for TPCN1 (p = 0.018), TPCN2 (p = 0.001) and IP_3_R1 (p = 0.035) was also increased relative to CTL (Table 3 and Figs. 3 and 4).

In CM-specific groups, mRNA expression was increased relative to CTL(n = 6): in ICM (n = 16), TPCN2 (p = 0.008); in DCM (n = 20), TPCN1 (p = 0.015) and TPCN2 (p = 0.001). These results were confirmed at the protein level: TPCN1 expression was increased significantly (p = 0.002) in DCM (n = 13) relative to CTL (n = 4) and TPCN2 expression was increased significantly in ICM (n = 8; p = 0.016) and DCM (n = 6; p = 0.031) relative to CTL (n = 4) (Table 3 and Fig. 3).

### CM-specific Correlations in Gene Expression (Table 4)

Having found specific CM-dependent increases in expression of four genes (CD36, HFABP, PPARA and PGC1A) involved in FA metabolism and HF-related increases in three Ca^2+^-handling genes (TPCN1, TPCN2 and IP_3_R1), we next compared gene expression levels by calculating Spearman correlation coefficients within CTL, ICM (n = 16) and DCM (n = 20) groups; any correlation with p<0.05 in CTL and p<0.01 in ICM and DCM was selected for further consideration. Of the 17 distinct gene pairs found to be correlated, 14 were positive (indicating that expression of both genes increased or decreased) and 3 were negative (indicating that expression of one gene increased while the other decreased) (Table 4).

**Table 4:** Spearman correlations in myocardial gene expression, in control and cardiomyopathy groups.

Gene	CD36	HFABP	CPT1B	LCAD	PGC1A	IP3R1	TPCN1	CHOP
PPARA	0.74**(2)	0.89*(1)		0.71**(3)	0.63**(2)	0.83*(1)	0.78**(2)	−0.83*(1)
	0.93**(3)				0.71**(3)			
CD36			0.89*(1)	0.62**(3)	0.68**(2)		0.67**(2)	
					0.60**(3)			
HFABP						0.83*(1)	0.94**(1)	
IP3R1					0.71**(3)			−0.83*(1)
TPCN1								0.64**(3)
TPCN2								−0.66**(2)

Numerical values indicate strength (Spearman ‘r’) and asterisks level of significance (*p<0.05, **p<0.01) of correlations in expression between genes in three groups: control (1, n = 6); ischaemic cardiomyopathy (2, n = 16); dilated cardiomyopathy (3, n = 20). ‘r’ values were positive except for three correlations with CHOP.

### *CTL (n = 6)*

In terms of correlations between genes involved in FA metabolism, CD36 was correlated with CPT1B (r = 0.89), and HFABP and PPARA with each other (r = 0.89). There were also correlations between FA metabolism genes and Ca^2+^-handling genes: HFABP and PPARA were each correlated with IP_3_R1 (r = 0.83 in each case), while HFABP was correlated with TPCN1 (r = 0.94). PPARA and IP_3_R1 each had negative correlations with CHOP (r = −0.83 in each case). Thus, in CTL there were 7 correlations among the genes that we studied, all of which had high ‘r’ values, indicating statistical reliability (Table 4).

### *ICM (n = 16)*

There were 6 correlations among the genes that we studied, all of which were distinct from those found in CTL (Table 4). CD36 was correlated with PPARA (r = 0.74) and PGC1A (r = 0.68), while PPARA was also correlated with PGC1A (r = 0.63). Both CD36 and PPARA were correlated with TPCN1 (r = 0.67 and r = 0.78, respectively). TPCN2 was negatively correlated with CHOP (r = −0.66).

### *DCM (n = 20)*

CD36 was correlated with PPARA, LCAD and PGC1A (r = 0.93, r = 0.62, and r = 0.60 respectively), while PPARA was also correlated with LCAD and PGC1A (r = 0.71 in each case). Finally, in this group CHOP showed its only positive correlation with the genes that we studied, with TPCN1 (r = 0.64). Thus, while we also found 6 correlations in this group which were distinct from those of CTL, three were common to and three distinct from ICM (Table 4).

## Discussion

This study investigated HF-related alterations in ventricular myocardial expression of genes along the PPARA- PGC1A-HFABP-β-oxidation pathway. In human HF of DCM but not ICM aetiology we found significant increases in expression over CTL levels of genes regulating FA uptake (CD36/FAT) and intracellular transport (HFABP). The high levels of significance found at the RNA level together with confirmation at the protein level show that all the groups were statistically reliable. Furthermore, comparison of the two aetiological groups with each other showed that CD36, PPARA and PGC1A were all significantly more highly expressed in DCM than ICM. Interestingly, expression of all three genes was correlated in both DCM and ICM, but not in CTL.

As PPARA is an important regulator of HFABP and CPT1B, and PGC1A co-activates PPARA and regulates β-oxidation [6], [12], [13], [23], our results suggest that there is a general up-regulation of the PPARA- PGC1A-HFABP-β-oxidation pathway in HF of DCM but not ICM aetiology. Our findings with PPARA are consistent with those of Schupp and colleagues [15] who found that LV myocardial expression of PPARA was increased at both mRNA and protein levels in 16 DCM patients compared with 15 CTL. In endomyocardial septal tissue from patients with HF of hypertrophic non-DCM aetiology no differences were found in PPARA mRNA expression; intriguingly, expression of the native protein was decreased but truncated protein increased in the same non-DCM group [16]. Although our tissue may have some different characteristics from those studied by Goikoetxea and colleagues [16], our results are in general agreement: compared with CTL, our non-DCM tissue did not show increased expression of PPARA either for mRNA or native protein; we did not study expression of the truncated form of the PPARA protein. The only other report of altered PPARA expression in human HF has also shown mRNA to be unaffected, although native protein expression was decreased; however that study involved only five patients with apparently undefined HF aetiology [14].

Similar to our findings, Karamanlidis and colleagues have also recently reported that expression of neither PPARA nor PGC1A is altered at the mRNA level in failing left ventricular myocardium when HF taken as a whole [45]. Notably, that study did find a substantial increase in protein expression for PGC1A in the HF group as a whole (n = 23) over non-HF (n = 19), although it did not find differences in either mRNA or protein levels between the two CM groups that made up HF (ICM (n = 9) and non-ICM (n = 14)). Other studies using human myocardium samples from different populations of end-stage heart failure patients have found increased [22], [46] or decreased mRNA expression in HF for PGC1A [46], [47]. Due to the variability that exist in the patient population between the different studies, differences in the therapies followed by the patients (some authors have proposed that angiotensin-converting enzyme inhibitors have a direct effect on cardiac remodelling and energy metabolism [48]) or in the clinical characteristics of the individuals (specially metabolic disorders such as diabetes; to note, we have selected for this study only non diabetic patients) could account in part for those controversial results.

Using experimental animal models of heart failure, other authors have reported unchanged or increased myocardial gene and/or protein expression levels of PGC1A and PPARA in rats [49], [50]; however, these results, obtained in rodents in which heart failure was induced after three weeks of aortic banding or by feeding for 17 weeks with high salt diet, are hardly comparable with those obtained in human failing myocardium.

Due to the limited amount of human tissue available to us, we did not study protein expression for genes in which mRNA levels were unchanged (i.e. in HF as a whole). However, having found that PPARA mRNA levels were significantly greater in DCM than ICM, we confirmed that at the protein level. Thus, compared with Karamanlidis and colleagues [45], we have found an additional CM-related alteration in PPARA expression. Moreover, our CTL and DCM groups were more strictly defined than the respective non-HF and non-ICM groups used in that study [45]. It should also be noted that although our CTL group comprised 6 individuals, our data-set from them was statistically reliable, as shown by several highly significant alterations in expression between CTL and various HF groups, as well as the tight Spearman correlations obtained within the CTL group. The same series of patients and donors has, moreover, been used in recently published studies [51], [52].

Our study did not show HF-related alterations in mRNA expression of either of the mitochondrial transport gene CPT1B or the β-oxidation gene LCAD, which are downstream of PPARA and PGC1A. This is in agreement with a previous study [45]. Other studies collectively show several discrepancies, with HF-related increases [18] or decreases in mRNA expression of CPT1B [46], and either unchanged mRNA [18] or decreased LCAD protein [20], [22], [46]. It should be noted that these studies have involved smaller numbers of patients of defined [22], non-defined [20], or mixed [18], [46] HF aetiology.

Two previous studies have analyzed the expression of CD36 in human failing myocardium: Pohl et al. [53] found no differences in CD36 expression in the plasma membranes of cardiomyocytes in patients with dilated cardiomyopathy, whereas Uray et al. [54] showed a transcriptional upregulation of CD36 linked to reverse remodelling in end-stage heart failure patients. Ours is, however, the first report of CD36 expression being increased in human HF in the absence of diabetes mellitus, or analysed in terms of ICM or DCM, but our results, taken together with the data for PPARA, PGC1A and HFABP and previous reports of increased FA levels perturbing mitochondrial function [24], [26]–[28] could make sense in one or more ways. In mouse myocardium, over-expression of PPARA has been shown to lead to over-expression of CD36 [55], and CD36 contains a PPARA-response element [56]. However, this effect might be indirect in cardiac [57], [58] and skeletal muscle [59]. Thus, CD36 could have been up-regulated at the sarcolemma and therefore account in terms of FA transport for the increases we observed in those intracellular components of the FA metabolism pathway. Alternatively or as well, CD36 might have been up-regulated at the mitochondrial membrane. Due to the limited amount of tissue available, we could not carry out cellular fractionation experiments to test these possibilities. Intriguingly, in a hypertrophic group of patients Heather and colleagues [60] have recently found a positive correlation between HFABP and CD36 at the protein level.

To summarise, this is the first time that CM-related diabetes mellitus independent differences have been reported in expression of genes involved in FA uptake, intracellular transport and oxidation in the same patients with failing human myocardium. It is notable that we also found potential alterations in the mutual regulation of some of these genes. Thus, in CTL CD36 was positively correlated with CPT1B, the principal mitochondrial membrane FA transporter, whereas in DCM it was correlated instead with the intra-mitochondrial β-oxidation enzyme LCAD. In ICM, by contrast, CD36 was correlated with neither of those mitochondrial factors but instead with the nuclear factors PPARA and PGC1A. HFABP was correlated only with PPARA and only in CTL. Taken together, these data suggest that regulation of CD36, HFABP and PPARA may differ not only in HF- but in CM-specific ways. On the other hand, there could also be alterations in gene regulation that are HF-related but not necessarily CM-specific, as seen by the positive correlations we found between CD36, PPARA and PGC1A in both DCM and ICM but not CTL.

We also found HF-related increases in expression of three genes involved in intracellular Ca^2+^ handling. Our increases in expression of IP_3_R1 in HF as a whole are in agreement with those that have previously established increased IP_3_R expression to be a general mechanism underlying changes in Ca^2+^-signalling in heart disease [33]. Interestingly, we also found highly significant HF-related increases in gene expression of the novel endo-lysosomal Ca^2+^ channels TPCN1 and TPCN2, recently identified as endo-lysosomal Ca^2+^ channels and components of the receptor for NAADP, the most potent intracellular Ca^2+^ mobiliser known [38], [39]. The up-regulation of both TPCN1 and TPCN2 that we found in HF could be related to the results of other studies showing increased expression of ryanodine receptors (RyR) in failing human myocardium [61], [62]. TPCN2, moreover, had similar levels of significance in all HF groups that we analysed, regardless of CM, therefore suggesting for the first time that TPCN2 up-regulation could also be involved in a general mechanism underlying heart failure, at least with ICM and DCM aetiology.

TPCN1 expression, on the other hand, while clearly increased in HF as a whole also tended towards a CM-difference. Thus, TPCN1’s increase in expression over CTL levels was statistically significant in DCM for both transcript and protein, whereas in ICM the transcript and the protein levels tended towards an increase that did not reach statistical significance. However, pertinent to a possible role in ICM, TPCN1 expression was correlated with that of CD36 and also of PPARA in ICM only. Indeed, those were the strongest (highest r values) and most significant HF-related correlations found between any Ca^2+^-handlers and any FA pathway gene in all HF groups that we studied (not all correlation data are shown). In contrast, in CTL, HFABP was strongly correlated with both IP_3_R1 and TPCN1, and PPARA with IP_3_R1.

The current data provide the first evidence that the endo-lysosomal system might be involved in cardiomyopathic alterations in Ca^2+^ signalling. It is already known that in failing myocardium IP_3_R from the ER becomes more highly expressed at the junctional sarcoplasmic reticulum (SR) where RyR is located, causing RyR to become sensitized and increasing diastolic Ca^2+^ levels and other Ca^2+^ transients characteristic of human heart failure [36]. In other systems, the central role of lysosomes is now becoming recognised. TPCNs having been shown to trigger global Ca^2+^ release by recruiting Ca^2+^-induced Ca^2+^-release (CICR) at lysosomal-ER junctions and to regulate plasma membrane excitability by the targeting of Ca^2+^-release from sub-plasma membrane stores, thereby regulating plasma membrane Ca^2+^-activated channels (recently reviewed by Galione [63]). Based on those studies and our current data, therefore, it may be hypothesised that in failing myocardium TPCN1 and/or TPCN2 are involved in altered distribution of Ca^2+^ to the SR and thereby to changes in membrane excitability that are known to occur in failing human heart [64].

### Limitations of the Study

1) Due to the limited amount of human tissue available (and the relatively large number of genes we investigated) we could not carry out protein level expression analyses for all genes, comparisons in gene expression with glucose transporters, or functional studies. 2) The cardioplegic solution used in the transplant protocols to preserve the condition/function of the hearts can decrease cardiac energy metabolism.

### Conclusions

In conclusion, using relatively large numbers of failing myocardial samples of ICM or DCM aetiology, we have found DCM-specific increases in expression of several important genes in the FA uptake and β-oxidation pathway. We have also found HF-related increases in expression of the novel endo-lysosomal Ca^2+^ handlers, TPCN1 and TPCN2; while TPCN2 was increased in all HF groups that we analysed, TPCN1 showed potential CM-specificity which merits further investigation. Finally, several of the genes investigated were found to be correlated, with patterns that differed between CTL and HF groups, suggesting HF- and CM-related alterations in the regulation of genes involved in FA and Ca^2+^myocardial metabolism. We therefore propose that pharmacological modulation of FA metabolism in HF as advocated by Opie [1] and Lionetti [2], among others, should be CM-specific in design, in particular for idiopathic DCM in which FA uptake and ß-oxidation are increased with respect to ICM.
